# Valorizing Tea Waste: Green Synthesis of Iron Nanoparticles for Efficient Dye Removal from Water

**DOI:** 10.3390/antiox13091059

**Published:** 2024-08-30

**Authors:** Cristina Rodríguez-Rasero, María F. Alexandre-Franco, Carmen Fernández-González, Vicente Montes-Jiménez, Eduardo M. Cuerda-Correa

**Affiliations:** Departamento de Química Orgánica e Inorgánica, Facultad de Ciencias, Universidad de Extremadura, Avenida de Elvas s/n, 06006 Badajoz, Spain; cristinarr@unex.es (C.R.-R.); malexandre@unex.es (M.F.A.-F.); mcfernan@unex.es (C.F.-G.); vmontes@unex.es (V.M.-J.)

**Keywords:** tea waste, polyphenols, valorization, nZVI, methylene blue, methyl orange, orange G

## Abstract

This study explores the valorization of tea leaf waste by extracting polyphenols through reflux extraction, subsequently using them to synthesize zero-valent iron nanoparticles (nZVI). The in situ generated nanoparticles, when combined with fixed amounts of hydrogen peroxide, facilitated the removal of various dyes (methylene blue, methyl orange, and orange G) via a hetero-catalytic Fenton process. The iron nanoparticles were thoroughly characterized by gas adsorption of N_2_ at 77 K, scanning electron microscopy (SEM), Transmission Electron Microscopy (TEM), FT-IR spectroscopy, X-ray diffraction (XRD), and thermal analysis, including thermogravimetric analysis (TG) and temperature-programmed reduction (TPR). A statistical design of experiments and response surface methodology were employed to analyze the influence of polyphenol, Fe(III), and H_2_O_2_ concentrations on dye removal efficiency. The results demonstrated that optimizing the operational conditions could achieve 100% dye removal efficiency. This study highlights the potential of nZVI synthesized through eco-friendly methods as a promising solution for water decontamination involving diverse model dyes, thus contributing to sustainable waste management and environmental protection.

## 1. Introduction

Water is a natural, scarce, and indispensable resource essential for human life and environmental sustainability. It is crucial in both qualitative and quantitative terms within ecosystems. Despite the planet having 36 × 10^15^ m^3^ of drinking water available, which represents only 1% of the total existing water, much of it is unusable due to environmental degradation and pollution. Although the freshwater supply is theoretically sufficient for global needs, it is unevenly distributed geographically and temporally, leading to water scarcity in certain regions., causing serious health issues for a significant portion of the global population.

There is a direct relationship between the standard of living in a society and the amount of waste generated, with higher industrial water use in higher-income countries (10% in low- and middle-income countries versus 59% in higher-income countries). Adequate water resources are vital for poverty reduction, economic growth, and environmental sustainability, affecting food and energy security, human health, and the environment. Poor water quality impedes economic development and poses health risks, especially in developing countries where chemical pollution from industrial and agricultural discharges is prevalent [1]. Industrialization has led to significant aquatic environmental degradation, with developing countries less aware of environmental issues compared to developed countries. While nature can self-regenerate from small pollution amounts, excess pollution exceeds its capacity.

One major environmental concern is pollution from persistent organic pollutants (POPs), which resist photochemical, chemical, and biochemical degradation, resulting in long half-lives in the environment. POPs have been detected in rivers, lakes, oceans, and even drinking water worldwide [2]. Although the carcinogenic, mutagenic, and bactericidal properties of most POPs are not fully understood, their removal from water is crucial to avoid potential toxic effects on living organisms, including humans [3].

For decades, research has focused on chemical pollutants regulated by legislation, such as polycyclic aromatic hydrocarbons (PAHs), polychlorinated biphenyls (PCBs), and dioxins. Recently, more sensitive analytical methods have revealed the presence of emerging pollutants, including pharmaceuticals, personal care products, detergents, and dyes [4]. Emerging pollutants, despite not being regulated yet, are concerning due to their continuous introduction into the environment and potential harmful effects. Synthetic dyes, particularly, are discharged in large quantities from industrial activities and pose significant environmental risks due to their stability and resistance to degradation [5,6].

Orange G, a toxic synthetic azo dye, and methylene blue (MB), used in various industries and medical applications, are notable pollutants. Methyl orange (MO), another azo dye, is used in pharmaceuticals and as a pH indicator. These dyes are resistant to conventional treatments, necessitating advanced methods.

Advanced oxidation processes (AOPs) are promising alternatives for water treatment. These processes use hydroxyl radicals to achieve pollutant mineralization, eliminate odors, disinfect water, and convert organic pollutants into less toxic compounds. AOPs, including Fenton and Fenton-like processes, use hydrogen peroxide and iron salts to generate hydroxyl radicals. The Fenton process is effective but costly and requires post-treatment removal of iron salts. The Fenton-like process, using cheaper Fe(III) salts, still faces challenges with homogeneous catalysis.

Heterogeneous catalysis, involving zero-valent iron nanoparticles (nZVI), offers a solution. Nanoparticles can be synthesized by reacting metal salts with conventional reductants, such as sodium boron hydride:

4Fe^3+^_(aq)_ + 3BH_4_^−^_(aq)_ + 9H_2_O_(l)_ → 4Fe^0^_(s)_ + 3H_2_BO_2_^−^_(aq)_ + 12H^+^_(aq)_ + 6H_2(g)_

Conventional reductants pose environmental and health risks, prompting the exploration of greener alternatives using plant extracts. Green synthesis of nanoparticles uses plant extracts with high antioxidant power, such as polyphenols, to reduce metal ions. This environmentally friendly approach stabilizes nanoparticles, preventing aggregation and allowing control over nanoparticle size without surfactants or stabilizers. The synthesis of nZVI with flocculation function also represents a key objective in green synthesis, as it combines environmental benefits with practical applications in water treatment [7].

The green synthesis of nanoparticles addresses the high cost and environmental damage associated with conventional methods. Plant extracts, rich in antioxidants, reduce metal ions to form nanoparticles. These plant-based methods stabilize nanoparticles and control their size, offering a bioproduction alternative. Using plants like green tea, which contain polyphenols, reduced sugars, nitrogenous bases, and amino acids, can induce nucleation and prevent particle aggregation. Green tea, one of the most widely consumed beverages globally, is recognized for its rich content of polyphenols, which are powerful antioxidants [8] and have the capacity to slow down or prevent oxidation of other molecules [9,10]. The main antioxidant compounds known to be present in tea include several catechins and catechin gallates, gallic acid, theaflavin and some theaflavin gallates, and theogallin [11]. Green tea has been found to have the highest antioxidant activity, followed by white, black, red, and other teas such as yerba mate and rooibos [12]. Tea has been reported to prevent and manage chronic diseases such as cancer, diabetes, obesity, and cardiovascular diseases, with the antioxidant capacity of tea being responsible for these health benefits [13]. The antioxidant properties of tea polyphenols have been extensively studied for their health benefits, including anti-inflammatory [14,15,16], anti-carcinogenic [17,18], and cardioprotective effects [19]. Beyond their health benefits, the extracted polyphenols hold promise for diverse applications, including nutraceuticals, food preservation, cosmetics, and environmental remediation, aligning with global sustainability goals [20]. For instance, epigallocatechin gallate (EGCG), the most abundant component of green tea polyphenols, has been shown to inhibit soil nitrification, affecting microbial communities and the soil nitrogen cycle [21]. Research on the antibacterial mechanism of tea polyphenols has led to their potential use as natural disinfectants for drinking water, offering a sustainable solution to conventional disinfection products [22].

Due to the extensive consumption of tea worldwide, huge amounts of tea waste are produced every day. Tea residues from the tea-making process have various potential uses. Waste tea has been explored as an alternative source of polyphenols to achieve economic added value [23]. Also, it can be used as a material for anaerobic fermentation biogas production, with studies showing significant biogas potential and optimal process conditions for degradation rates [24]. Tea proteins extracted from residues have functional properties suitable for food supplements and various food manufacturing applications, such as gelation capacity for dairy products, jellies, and bread, as well as antioxidant and antihypertensive bioactivities [25].

This work investigates the green synthesis of nZVI using green tea waste extracts for the degradation of orange G, methylene blue, and methyl orange dyes through the Fenton-like process. Green tea waste extracts, rich in polyphenols, serve as reducing agents to produce nZVI, which then acts as a heterogeneous catalyst in dye degradation. This method offers an environmentally friendly and cost-effective solution for treating dye-contaminated water, addressing the limitations of conventional water treatment methods and contributing to environmental sustainability.

## 2. Materials and Methods

### 2.1. Sample Acquisition and Conditioning

The sample used in this study comprises tea leaves powder obtained from green tea sachets, which were purchased from a local store. The starting material was obtained from a controlled and consistent source, with all tea bags coming from the same brand and lot number to ensure minimal variation in the polyphenol content. The tea sachets were first used to prepare tea infusions in a domestic setting. After the preparation of the tea, the sachets were carefully opened, and the post-extracted tea waste was collected. This tea waste, which consists of the tea leaves powder remaining after the extraction of the infusion, was then subjected to a drying process in a laboratory oven at a controlled temperature of 40 °C. This drying process was continued until a constant weight was achieved, ensuring that all residual moisture was removed to avoid affecting subsequent analyses.

Once dried, the tea waste was allowed to cool to room temperature, and all the waste used in this study was blended before extraction to achieve a more uniform starting material. The dried tea waste was then carefully transferred into tightly closed containers. These containers were chosen to ensure that the samples were protected from exposure to humidity and other environmental factors that might compromise their integrity.

It is worth noting that the novelty of this study lies in the use of post-extracted tea waste, a commonly discarded by-product, as a valuable resource. By utilizing the tea leaves remaining after the preparation of tea infusions, this approach highlights an innovative and sustainable method for repurposing waste material. This not only reduces environmental impact but also leverages the antioxidant properties inherent in the tea leaves for further applications.

### 2.2. Extraction of Polyphenols from Green Tea Waste: Determination of the Polyphenols Content

The tea waste extract was prepared through reflux extraction, where 5 g of tea waste was boiled in 100 mL of distilled water at a temperature below 80 °C for 20 min. After extraction, the mixture was cooled to room temperature and filtered using a double vacuum filtration system to separate the solid residue.

The composition of the extract was qualitatively analyzed using methods described by Dobrinčić et al. [NO_PRINTED_FORM] [26]. Initially, samples were filtered through 0.45 μm nylon membrane filters. An HPLC system (Agilent Technologies HPLC 1260 Series) (Agilent Technologies, Santa Clara, CA, USA) equipped with a UV/Vis-Photo Diode Array Detector (DAD) and a Luna C18 column (3 μm, 150 mm × 2 mm, 100 Å, Phenomenex, Torrance, CA, USA) was used to separate and identify polyphenols. Two mobile phases were used: 0.1% formic acid in water (A) and 0.1% formic acid in methanol (B). The separation was achieved with the following gradient intervals: 0–3 min, 10% B; 3–30 min, 50% B; 30–40 min, 60% B; 40–45 min, 60% B; 45–50 min, 100% B; and 50–60 min, 10% B. The flow rate was 1 mL·min^−1^, the column temperature was set at 30 °C, and the injection volume was 20 μL. Detection was performed at λ = 280 nm.

Given the mixture of polyphenolic compounds in the extract, total polyphenol content was determined using the Folin–Ciocalteu assay [27]. This assay measures the reaction between phenolic compounds and Folin’s reagent at basic pH, achieved by adding Na_2_CO_3_. Folin’s reagent, consisting of sodium tungstate and sodium molybdate in phosphoric acid, forms a yellow phosphomolybdotungstic acid complex in an acidic medium, which turns blue upon reduction by phenolic groups. The absorbance of the resulting complex was measured using a Shimadzu UV-1800 spectrophotometer (Shimadzu, Tokyo, Japan).

Quantification was based on a gallic acid standard line (see Table S1 and Figure S1, Supplementary Materials), with polyphenol concentration expressed as gallic acid equivalents. Various concentrations (4.0 to 20.0 mg/L) of gallic acid standards were prepared from a 196 mg/L stock solution. Each solution was mixed with 0.5 mL of Folin’s reagent and 10 mL of 7.5% Na_2_CO_3_, then diluted to 25 mL with ultrapure water and left in the dark for 1 h before measuring absorbance at 760 nm against a blank.

For the green tea extract, volumes of 50, 75, and 100 μL were taken, mixed with Folin’s reagent and Na_2_CO_3_, and diluted to 25 mL with ultrapure water. After one hour in the dark, absorbance was measured at 760 nm.

### 2.3. Preparation of the Zero-Valent Iron Nanoparticles, nZVI, and Removal of the Dye: Influence of Variables

Nanoparticles were synthesized in situ using the procedure described below. For the sake of clarity, this procedure is schematically illustrated in Figure S2 (Supplementary Materials). Firstly, a dye solution (orange G, methylene blue, or methyl orange) with a fixed concentration of 100 ppm was placed in a beaker on a stirring plate equipped with a magnetic stirrer (depicted as 1 in Figure S2). The appropriate volume of tea extract was added to achieve the desired polyphenol concentration, and water was added to bring the total volume to 75 mL (number 2 in Figure S2).

A 25 mL solution of Fe(III) salt at the required concentration was then added dropwise to the mixture (number 3 in Figure S2), ensuring the final Fe(III) concentration matched the experimental matrix described in Table 1 and Table S2 (Supplementary Materials). The mixture was stirred magnetically at 500 rpm. Upon adding the Fe(III) solution, the formation of zero-valent iron nanoparticles (nZVI) was observed as the solution turned opaque and black. The mixture was allowed to react for 5 min with continuous stirring.

Subsequently, the appropriate amount of hydrogen peroxide was added (number 4 in Figure S2), and the system was stirred for an additional 15 min.

After the reaction period, a sample of the solution was extracted and centrifuged at 5000 rpm to separate the nanoparticles from the supernatant. The remaining dye concentration in the solution was then analyzed spectrophotometrically to calculate the percentage of dye elimination from the initial amount present.

### 2.4. Characterization of the nZVI

The samples prepared as described were subjected to a comprehensive characterization, including texture and morphology analysis, chemical composition examination, and thermal behavior assessment.

#### 2.4.1. Chemical Characterization and Crystal Structure

Surface chemistry analysis was conducted using FT-IR spectroscopy with the aid of a Perkin Elmer 1720 spectrophotometer (PerkinElmer, Waltham, MA, USA). Spectra were recorded in the wave number range between 400 and 4000 cm^−1^ with a resolution of 2 cm^−1^, each consisting of fifty scans. Disks for analysis were prepared using KBr as a dispersing agent and binder, with a sample-to-KBr ratio of 500:1 and a total mass of 200.5 mg.

The crystal structure was investigated using X-ray diffraction (XRD). Diffraction patterns were acquired with a Bruker D8 ADVANCE (Bruker, Billerica, MA, USA) diffractometer equipped with a PSD Vantec detector, within the 2θ range from 15° to 80°, with a step size of 0.02° and using Cu Kα radiation (λ = 1.54184 Å).

#### 2.4.2. Texture and Morphology

Textural characterization involved gas adsorption of N_2_ at 77 K, utilizing a semi-automatic Quantachrome Autosorb-1 (Quantachrome Instruments, Boynton Beach, FL, USA). For this, 0.15 g of nZVI sample was degassed at 250 °C for 12 h under a pressure below 10^−3^ Torr. The micropore volume (V_mi_) was calculated from the adsorption isotherms at a relative pressure (p/p°) of 0.10, where p° represents the standard pressure, which has not been modified over time. The mesopore volume (V_me_) was determined using the formula V_me_ = V_0.95_ − V_mi_, where V_0.95_ is the volume of nitrogen adsorbed at a relative pressure of 0.95. Both V_mi_ and V_me_ were expressed in liquid volumes, and the conversion factor was 1.543 × 10^−3^.

The specific surface area (S_BET_) was estimated using the Brunauer, Emmet, and Teller (BET) method [28], while the micropore volume (W_0_) was obtained using the Dubinin–Radushkevich equation [29].

Morphology was analyzed using scanning electron microscopy (SEM) and Transmission Electron Microscopy (TEM). Field-emission scanning electron microscopy (FESEM) images were acquired with a Jeol JSM-7800F Prime microscope (JEOL Ltd., Taiwan, China) equipped with an EDX analyzer for element mapping. High-resolution images were collected using a transmission electron microscope (TEM, Jeol JEM 1400) (JEOL Ltd., Taiwan, China). Samples were dispersed in water under sonication and deposited on a holey carbon grid.

#### 2.4.3. Thermal Analysis

Thermal analyses were conducted using a STA 449 F3 Jupiter-Netzsch thermobalance (NETZSCH, Selb, Bavaria, Germany), coupled to a QMS 403D Aëolos III-Netzsch mass spectrometer (NETZSCH, Selb, Bavaria, Germany). Temperature Programmed Reduction (TPR) assays were performed over a temperature range from 40 to 800 °C, with a heating rate of 5 °C/min under a N_2_:H_2_ (90:10) atmosphere with a total gas flux of 200 mL min^−1^. The evolution of gases, specifically H_2_O (*m*/*z* = 18) and CO_2_ (*m*/*z* = 44), was continuously monitored.

For Temperature Programmed Desorption (TPD) and Oxidation (TPO) analyses, the same experimental conditions were used, but with varied atmospheres and gas flows. TPD was conducted under a 100% N_2_ atmosphere with a gas flow rate of 100 mL min^−1^, while TPO was performed using an N_2_:O_2_ (80:20) atmosphere with a gas flow rate of 100 mL min^−1^.

### 2.5. Experimental Design

To investigate the impact of three operational variables, namely the concentrations of polyphenols, Fe(III), and H_2_O_2_, on the efficiency of dye removal, a factorial, composite, central, orthogonal, and rotatable (FCCOR) experimental design was employed. This design consisted of 8 factorial experiments, 6 axial experiments, and 9 replicates of the central experiments, resulting in a total of 23 experiments in the experimental matrix. Table S2 (Supplementary Materials) lists the coded and real values of the operational variables, as well as the set of 23 experiments that constitute the matrix. When experimental designs are used, it is of the utmost importance to adequately determine the operational intervals. In this case, in a previous paper by the same authors [30], the individual influence of the different variables was thoroughly studied, and the operational intervals were settled accordingly. Hence, the same operational intervals have been used here, with the only exception that in this case, the concentration of Fe(II) did not exert any remarkable influence on the removal efficiency of dyes and, thus, only Fe(III) was used as the iron precursor.

## 3. Results and Discussion

### 3.1. Determination and Quantification of Polyphenols in Green Tea Waste Extracts

**Figure 1:**
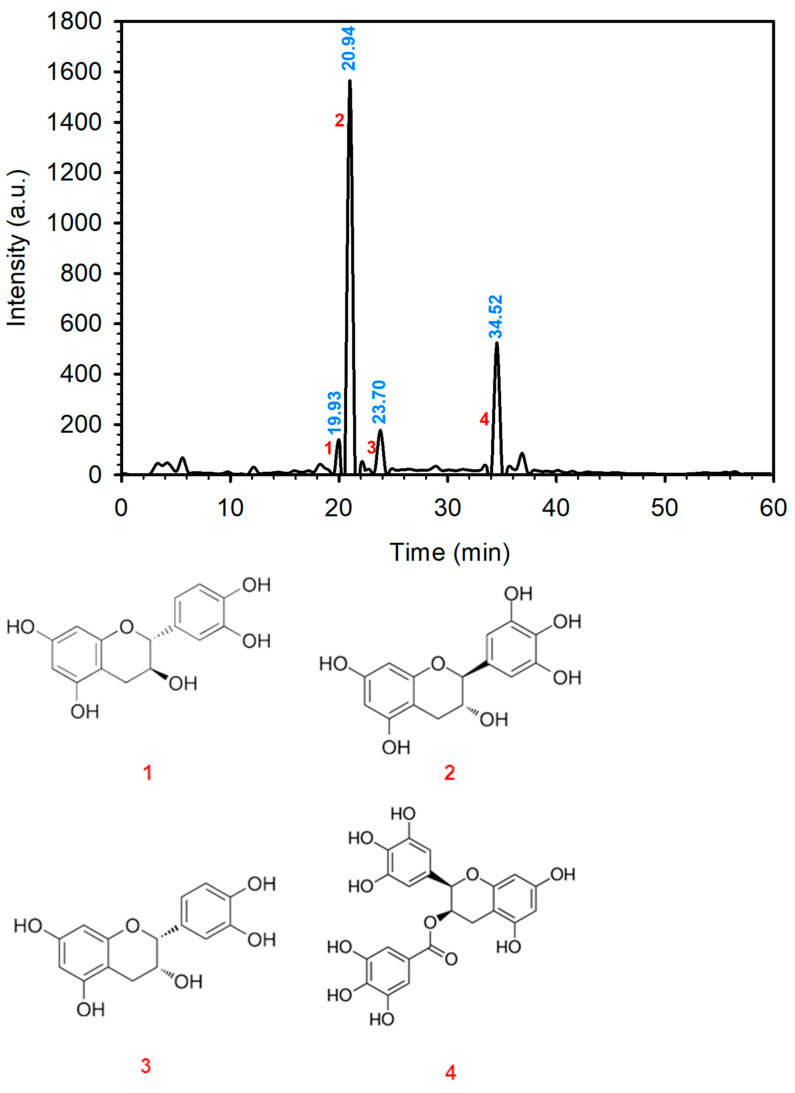
HPLC-DAD profile of green tea leaf waste (**top**) and structure of the primary polyphenols identified (**bottom**). **1.** Catechin; **2.** gallocatechin; **3.** epicatechin; **4.** epigallocatechin-3,5-digallate. Retention times are shown in blue.

The analysis of the polyphenol content of the tea waste extract is critical for the green synthesis of nZVI, as it makes it possible to establish the effectiveness of tea waste extract as a reducing agent in nZVI synthesis. The polyphenol content in the green tea waste was determined following the procedure described in Section 2.1. The HPLC chromatogram of the extract is presented in Figure 1 (top).

The peaks observed in the HPLC-DAD analysis were identified based on the existing literature [31,32,33,34]. Among the predominant polyphenols detected in the extract were gallocatechin and epigallocatechin-3,5-digallate, which is in good agreement with other studies previously reported in the literature [8,35]. Additionally, other polyphenolic compounds such as catechin and epicatechin were also identified. These corresponding peaks in the chromatogram have been labeled, and their chemical structures are depicted in Figure 1 (bottom).

As mentioned in Section 2.3, for the sake of simplicity and ease of comparison, the total polyphenols content in the green tea waste extract sample was quantified and expressed as gallic acid equivalents. The polyphenol content was found to be 1390 ± 67 mg of gallic acid equivalents per liter of tea waste extract, equivalent to 27,840 ± 1346 mg of gallic acid equivalents per kilogram of dry tea waste. It is worth noting that, while the antioxidant composition of waste tea residue extracts can vary significantly, the residual polyphenol content, though modest in economic value, plays a crucial role in facilitating green synthesis and enhancing environmental sustainability.

### 3.2. Preparation and Characterization of the Zero-Valent Iron Nanoparticles, n-ZVI

#### 3.2.1. Chemical Characterization and Crystal Structure

**Figure 2:**
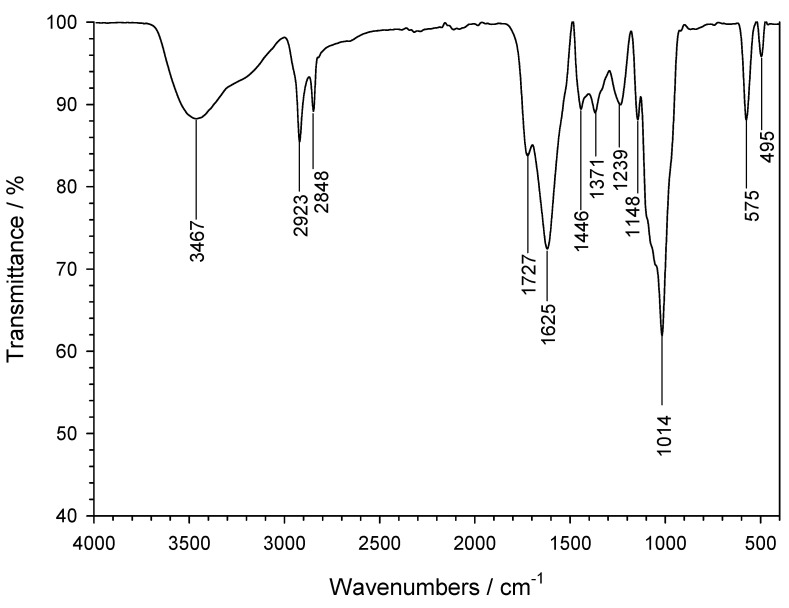
FT-IR spectrum of the nZVI sample.

An adequate characterization of the chemistry and crystal structure of the sample helps to demonstrate the successful synthesis of nZVI with desirable properties for environmental applications. With such an aim, the nZVI samples were first analyzed by FT-IR spectroscopy, as illustrated in Figure 2, where a wide variety of spectral features can be appreciated. Firstly, the bands centered around 495 and 1371 cm^−1^ are typical of Fe-O bonds in Fe_2_O_3_. Also, the band located at 575 cm^−1^ may be due to Fe=O bond stretching both in FeO or Fe_2_O_3_. Other bands, on the contrary, can be assigned to the polyphenolic compounds that are present in the green tea waste extract, as discussed earlier. For instance, the bands of variable intensity centered at 1014, and 1148 cm^−1^ can be due to in-plane vibrations of C-H bonds in aromatic rings. The band located at 1239 cm^−1^ is attributable to the stretching vibration of bonds between aromatic carbon atoms and -OH groups (arC-OH) that are commonly present in polyphenols. The band at 1446 cm^−1^ corresponds to the in-plane deformation vibration of -OH groups.

The bands centered at 1625 and 1727 cm^−1^ can be assigned to stretching vibrations of C=C bonds in aromatic rings and C=O bonds in phenolic esters, respectively. The bands that can be appreciated at 2848 and 2923 cm^−1^ are due to -CH_2_- stretching vibrations. In particular, the band centered at 2848 cm^−1^ is compatible with stretching vibrations in -O-CH_2_- bond in non-cyclic eters. Finally, the broad band centered at 3467 cm^−1^ is commonly associated with the presence of hydroxyl groups in polyphenols, although it is also compatible with the occurrence of -OH in FeOOH.

**Figure 3:**
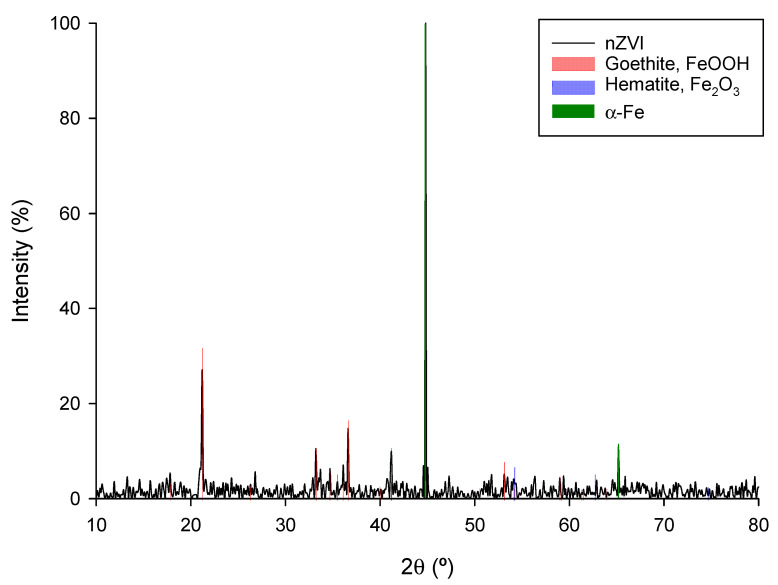
XRD pattern of the nZVI sample.

The samples in this section were subjected to X-ray diffraction analysis. Figure 3 displays the XRD pattern for the nZVI sample, which is consistent with the presence of crystalline FeO.

Notably, a prominent diffraction peak at 2θ = 44.85° corresponding to the lattice plane of (1 1 0), and a weaker peak at 2θ = 65.2° for the lattice plane of (2 0 0), confirm the generation of zero-valent iron nanoparticles. These nanoparticles result from the reduction of Fe^3+^ by the polyphenols found in the extract of green tea leaves. The XRD patterns, and the semi-quantitative analysis of the different crystalline phases present in the sample based on them (see Table 2) also reveal additional reflections corresponding to goethite (FeOOH) and hematite (Fe_2_O_3_), indicating that FeO can be partially re-oxidized by the aqueous media used during synthesis.

These findings align with previous literature reports [36,37,38,39].

This evidence, together with the results of the FTIR analysis, suggests that the nanoparticles may consist of a Fe^0^ core surrounded by a higher oxidation state iron oxide (Fe_2_O_3_) or oxohydroxide (FeOOH). This is in good agreement with other works previously reported in the literature [40,41,42,43].

#### 3.2.2. Texture and Morphology

An adequate analysis of texture and morphology is essential to understanding the physical characteristics of the synthesized zero-valent iron nanoparticles (nZVI). In this section, the surface texture, pore structure, and morphology of nZVI are examined.

**Figure 4:**
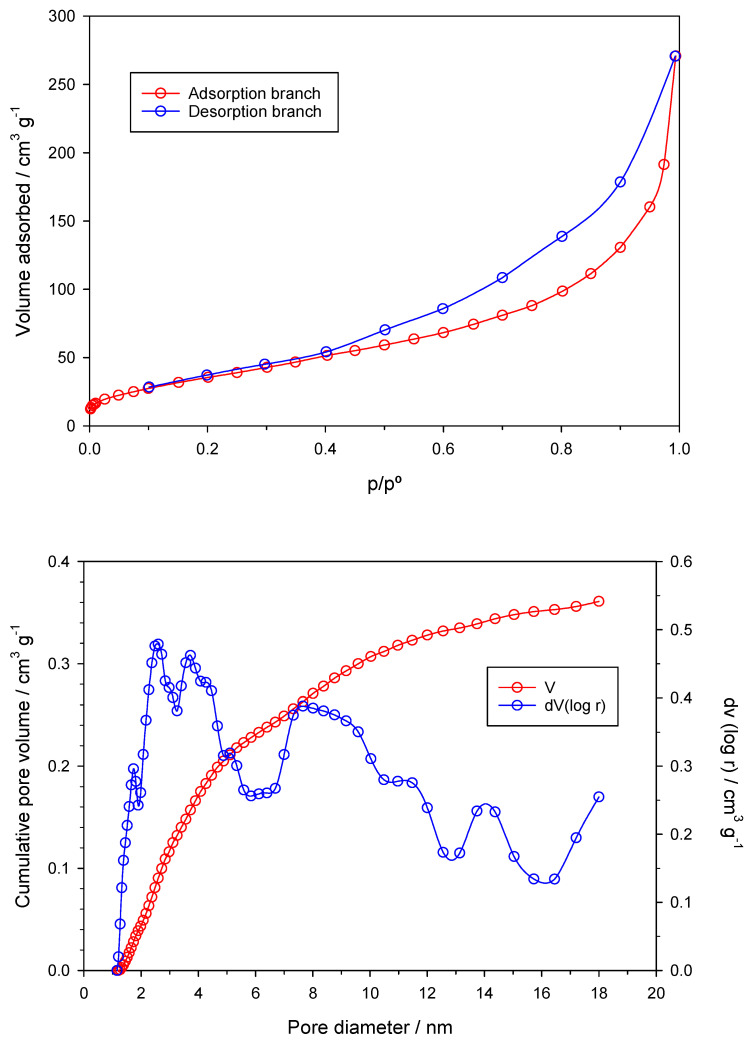
N_2_ adsorption isotherm at −196 °C (**top**) and pore size distribution (**bottom**) of the nZVI sample.

Figure 4 (top) depicts the N_2_ adsorption isotherm of the nZVI sample at −196 °C, revealing a notably high adsorption capacity, classified as Type IV according to the BDDT classification due to the observed hysteresis loop. This hysteresis, observed between relative pressure values around 0.1 and p/p° ≈ 1, is attributed to capillary condensation within mesopores, suggesting a wide pore size distribution, mainly in the mesopore range. The pore size distribution plot (Figure 4 (bottom)) confirms a well-developed mesoporosity with pore diameters ranging from 3 to 18 nm.

Numerical calculations support these findings, with the sample showing a low micropore volume of around 0.041 cm^3^·g^−1^ (V_mi_) or 0.045 cm^3^·g^−1^ (W_0_), and a significant contribution of mesopores, with a mesopore volume (V_me_) of 0.211 cm^3^·g^−1^ and an average pore diameter (APD) of 13.54 nm. The specific surface area (S_BET_) reaches 134 m^2^·g^−1^, notably exceeding previous literature values for nZVI samples. While most pristine nZVI samples in the literature exhibit S_BET_ values ranging from 5 to 61 m^2^·g^−1^ [44,45,46], some studies report higher values, reaching up to 143 m^2^·g^−1^ [47]. These values are similar to or slightly larger than those reported in this work. However, it is notable that these higher values were achieved through the reduction of Fe^3+^ with a conventional reductant like sodium borohydride, contrasting with the greener approach employed in this study.

The fractal dimension (D) serves as a roughness exponent, with Euclidean (ideal) surfaces assumed to have D equal to 2. In contrast, for irregular (real) surfaces, D may vary between 2 and 3, representing the degree of surface roughness and/or porous structure. The value of D here obtained (2.64) indicates a remarkably rough surface.

**Figure 5:**
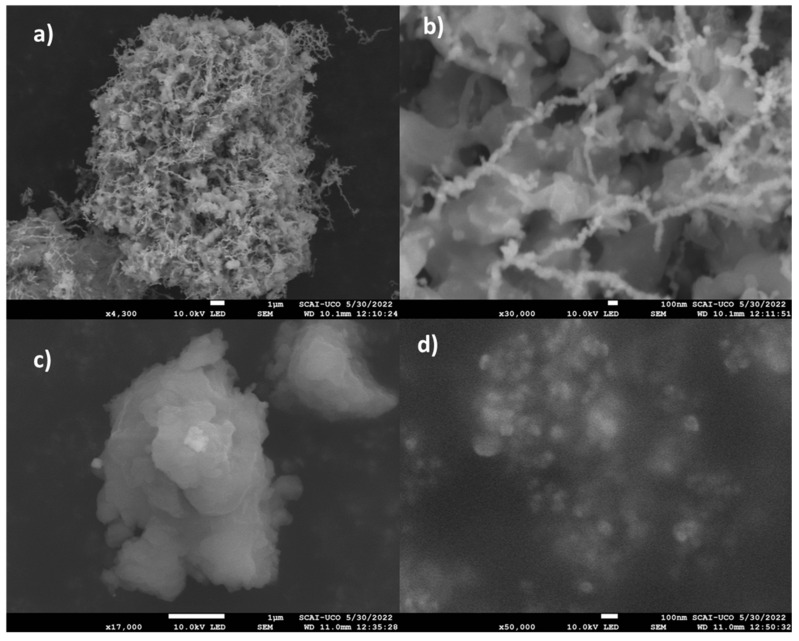
SEM images of nZVI samples prepared using NaBH_4_ (**a**,**b**) and tea waste extract (**c**,**d**) as the reducing agents.

The SEM images depicted in Figure 5 (and, at a larger size for the sake of clarity in Figure S3, Supplementary Materials) corroborate this latter assertion. It can be appreciated that nZVI tends to agglomerate, thus giving rise to a surface with a noticeable degree of irregularity. In fact, the morphology of the sample is coherent with the occurrence of irregular iron nanoparticles with a diameter around or below 50 nm that form aggregates. This results, on the one hand, in the irregular and rough aspect of the surface, and, on the other hand, in the generation of an inter-particle mesoporosity.

For comparison, nZVI samples were synthesized using sodium borohydride (NaBH_4_) as the reductant, following the same procedure described in Section 2.2. SEM images revealed that the nZVI produced using tea waste extract formed aggregates ranging from a few microns up to 100 microns. Similar patterns were seen in samples made with NaBH_4_, capturing clear images proved challenging due to exceedingly high electrostatic charge. At higher magnifications, nanoparticle aggregates, some around 100 nm or smaller, were visible. Two distinct structures were identified: interconnected nanoparticles forming substantial fibers, and larger, heterogeneous aggregates.

**Figure 6:**
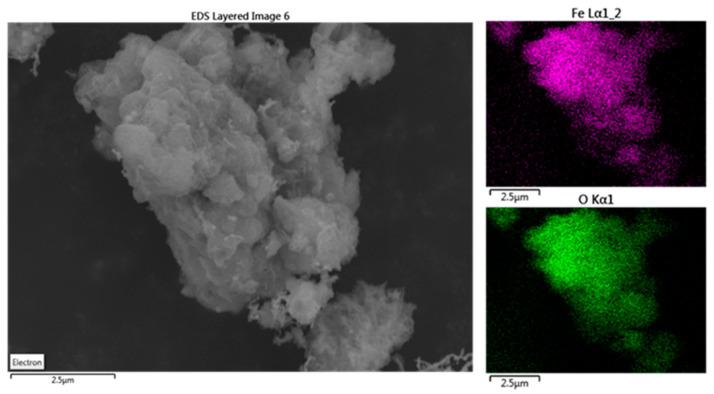
SEM images (**left**) and EDX mapping images ((**top right**) iron and (**bottom right**) oxygen) of the nZVI sample.

Based on SEM-EDX images (Figure 6), iron and oxygen exhibited even distribution without any noticeable concentration at the micron scale.

On the other hand, EDX mapping reveals the presence of other elements such as C, and, in significantly lower concentrations, Cl, P, and Na. The substantial presence of carbon and oxygen is coherent with the occurrence of carbonyl/carboxyl and phenolic moieties of polyphenols and reflects the presence of organic matter, corresponding to the dry residue of the polyphenols extract. Small amounts of essential oligo-elements such as phosphorus, chlorine, and sulfur are also detected, further supporting the presence of organic matter. This is in good agreement with other results previously reported in the literature [37,48,49,50]. However, this does not impede the catalytic activity of Fe^0^ in heterogeneous Fenton reactions, as the system remains under constant stirring, and any residual polyphenols depositing onto the nZVI particles are promptly removed. Only when the nanoparticles are filtered and dried does the remaining organic matter cover the nZVI.

**Figure 7:**
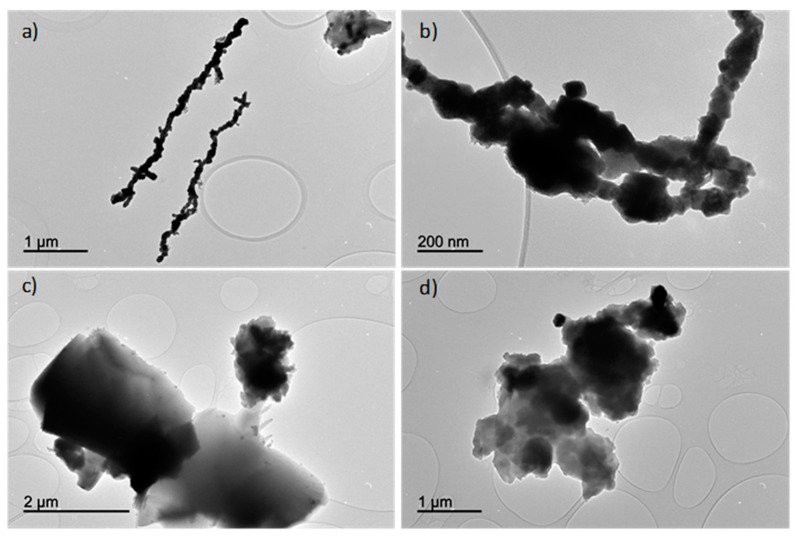
TEM images of nZVI samples prepared using NaBH_4_ (**a**,**b**) and tea waste extract (**c**,**d**) as the reducing agents.

The heterogeneity of particle sizes and shapes is apparent in the TEM images (Figure 7). Generally, nanoparticles synthesized using tea waste extract displayed particle sizes exceeding 500 nm, exhibiting a diverse array of shapes, including rounded and rectangular forms. In contrast, nanoparticles produced with sodium borohydride were smaller, falling within a range of 50 to 1000 nm.

#### 3.2.3. Thermal Analysis

**Figure 8:**
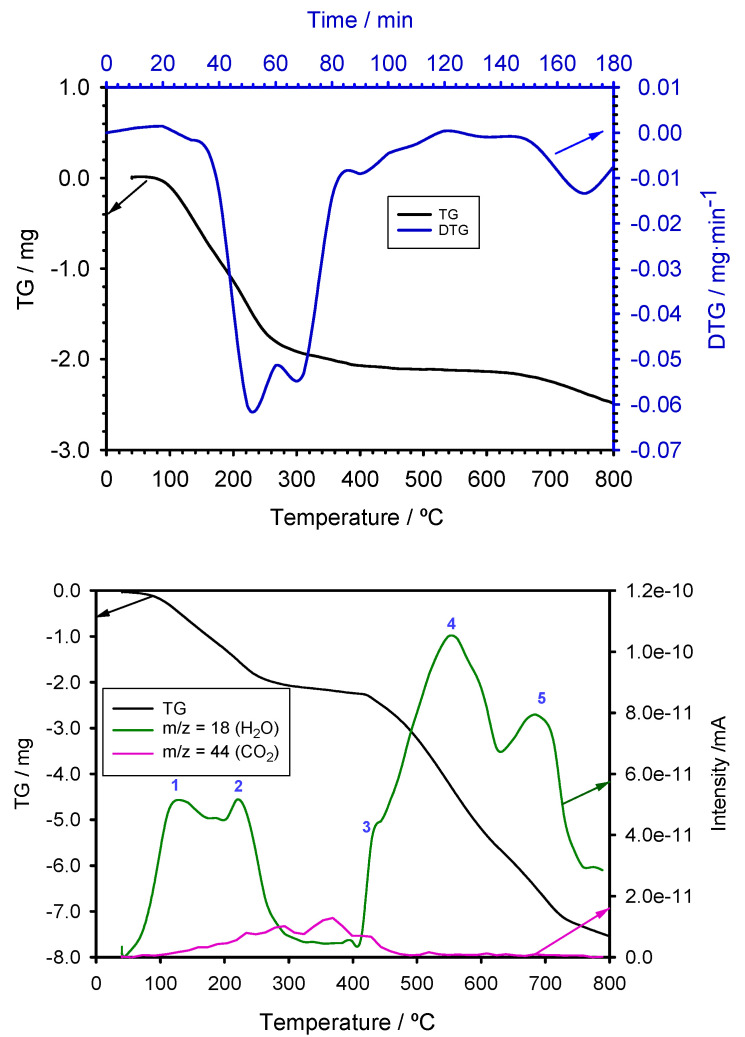
TG/DTG (**top**) and TPR (**bottom**) analyses of the nZVI sample. H_2_O and CO_2_ release profiles are shown in the TPR plot.

A thorough analysis of thermal behavior is crucial for understanding the stability and reduction mechanisms of the synthesized zero-valent iron nanoparticles (nZVI). In this section, the thermal properties of nZVI are assessed through techniques like thermogravimetric analysis (TG) and temperature-programmed reduction (TPR), providing insights into their thermal stability and the reduction processes they undergo. Figure 8 (top) illustrates the TG-DTG curve corresponding to the nZVI sample prepared using green tea waste extract as the reducing agent. The plot reveals distinct mass loss stages. Initially, a subtle effect is observed, starting around 95 °C and continuing until approximately 140 °C. This initial stage can be attributed to the removal of residual moisture that might be present in the nZVI sample. At ~160 °C, a second and much more pronounced weight loss phase begins. This stage, centered at 230 °C, is indicative of the dehydration of goethite, as explained by the following reaction:

(1) $$2FeOOH\to {Fe}_{2}O_{3}+H_{2}O$$

Following this, a third mass loss effect starts at around 270 °C and is centered at 300 °C. This observation aligns well with the onset of pyrolysis of the organic matter initially present in the sample, namely residues of the green tea waste extract. This pyrolysis process remains relatively intense until approximately 650 °C. Subsequently, the pyrolysis process appears to accelerate, leading to a noticeable increase in the slope of the TG plot beyond 650 °C.

The TPR profile of the partially oxidized iron nanoparticles, presented in Figure 8 (bottom), elucidates the reduction behavior when subjected to hydrogen as the reducing agent. The water release analysis reveals five distinct peaks, corresponding to different stages of sample reduction. CO_2_ evolution is also shown.

Initially, the desorption of surface-adsorbed water is observed at approximately 130 °C, indicating the removal of moisture from the nanoparticle surface. This is a characteristic feature in TPR profiles, reflecting the evaporation of physically adsorbed water molecules. Subsequently, at around 225 °C, a peak corresponds to the dehydration of iron oxohydroxide (FeOOH), aligning with the previously mentioned Reaction (1).

A significant increase in water release is noted between 410 and 425 °C, indicative of the initial reduction of hematite (Fe_2_O_3_) to magnetite (Fe_3_O_4_), described by the reaction:

(2) $$3{Fe}_{2}O_{3}+H_{2}\to 2{Fe}_{3}O_{4}+H_{2}O$$

The fourth peak, appearing at approximately 550 °C, represents the further reduction of magnetite to ferrous oxide (FeO), according to the reaction:

(3) $${Fe}_{3}O_{4}+H_{2}\to 3FeO+H_{2}O$$

Finally, the peak at around 690 °C signifies the reduction of ferrous oxide to metallic iron, with the reaction:

(4) $$FeO+H_{2}\to Fe+H_{2}O$$

Thus, the global process can be expressed as:

(5) $$2FeOOH+3H_{2}\to 2Fe+4H_{2}O$$

Additionally, the evolution of CO_2_ is observed within the temperature range of 150–450 °C, indicative of the thermal decomposition of organic matter remnants on the nanoparticle surface. Given that polyphenols were employed as the green reducing agent during nanoparticle synthesis, the presence of residual organic compounds is expected. The observed CO_2_ release is consistent with the degradation of these organic residues, and aligns well with the evidence that results from XRD and FTIR analyses, validating the hypothesis of a core-shell structure with a Fe(0) core surrounded by iron oxide and iron oxohydroxide.

The TG curve shows a continuous mass loss, correlating with the thermal events and reduction processes occurring within the sample.

### 3.3. Removal of Dyes: Influence of Variables

A comprehensive analysis of dye removal efficiency is essential for understanding the effectiveness of the synthesized zero-valent iron nanoparticles (nZVI). In this section, the removal of the three dyes under study is investigated, with a focus on how the different operational variables (i.e., the concentrations of polyphenol, Fe(III), and H_2_O_2_) influence the overall dye degradation performance. The efficiency of the synthesized nZVI in degrading methylene blue, methyl orange, and orange G dyes was evaluated using a hetero-catalytic Fenton process, whereas the response surface methodology optimized the operational parameters (concentration of polyphenols, Fe(III), and H_2_O_2_). Table 1 presents the removal efficiency results for methylene blue, methyl orange, and orange G across each of the 23 experiments comprising the experimental matrix. 

From the results summarized in Table 1, it can be easily concluded that most of the dyes are removed from water in an efficient manner by the nZVI. Maximum removal efficiencies of 94.4, 93.47, and 93.72% are achieved for MB, MO, and OG, respectively, whereas the minimum removal efficiencies are 35.31, 56.36, and 61.92%. Thus, it can be stated that, although the operational interval has been determined accurately, there is still some margin to optimize the removal of the three pollutants. The results of the experimental design will be discussed separately for each of the dyes in the following paragraphs.

#### 3.3.1. Removal of Methylene Blue

For methylene blue, polyphenols demonstrated a significant negative impact on removal efficiency, with an effect estimate of −25.4773 (see Table S3, Supplementary Materials). This indicates that higher concentrations of polyphenols reduce the effectiveness of methylene blue removal. Similar trends have been reported in the literature, where organic compounds have been shown to inhibit the catalytic activity of nZVI by occupying active sites [51,52,53], hindering the approach of contaminants to the nZVI surface due to steric effects [54] or blocking the contaminants reduction because of the formation of a less-conductive film that limits electron transfer from Fe^0^ to the pollutant molecules [55,56,57]. Conversely, hydrogen peroxide exhibited a strong positive influence (24.8959), enhancing the removal efficiency. This aligns with the well-documented role of hydrogen peroxide in generating hydroxyl radicals, which are crucial for dye degradation in Fenton-like processes [58,59,60,61]. Iron (III) had a slight negative effect (−4.99208), possibly due to excess iron leading to the scavenging of hydroxyl radicals [62,63]. In fact, the presence of an excess of Fe(III) results in a decrease in hydroxyl radicals production coupled with the formation of weaker reducing species such as peroxo (O_2_^−^·) and hydroperoxo (HO_2_^−^·) radicals. The interaction between polyphenols and iron (III) was notably negative (−12.93), suggesting a combined high concentration of these factors further decreases the efficiency.

The ANOVA results (Table S4, Supplementary Materials) indicate that five effects have *p*-values less than 0.05, confirming their statistical significance: the main effects of polyphenols and hydrogen peroxide, the quadratic terms for polyphenols and hydrogen peroxide, and the interaction between polyphenols and iron (III). The ANOVA analysis for the removal of methylene blue provides other statistical parameters to evaluate the model’s performance. The standard error of the estimate is 2.7281 (Table S3, Supplementary Materials). This value indicates the average distance that the observed values fall from the regression line. A lower standard error suggests that the observed values are closer to the predicted values, indicating a more accurate model. In this context, a standard error of 2.7281 is remarkably small, demonstrating that the model fits the data quite well. The mean absolute error is 1.50523. This parameter measures the average magnitude of the errors in the predictions, without considering their direction (i.e., the average of the absolute errors). A lower MAE indicates better predictive accuracy. In this case, an MAE of 1.50523 signifies that, on average, the model’s predictions deviate by approximately 1.5 units from the actual values, indicating a high level of accuracy. Finally, the Durbin–Watson statistic is 1.57596 with a *p*-value of 0.1627, which indicates that there is no significant autocorrelation in the residuals, suggesting that the model’s residuals are independent. These parameters collectively indicate that the regression model for methylene blue removal is robust and reliable, with low prediction errors and no significant autocorrelation, thereby validating the model’s assumptions and its predictive accuracy. These assertions are corroborated by the fact that the regression model revealed a strong fit (R^2^ = 94.56%), with the equation:

Removal %(Methylene blue) = 82.167 − 12.7386·[Polyphenols] − 2.49604·[Fe^3+^] + 12.4479·[H_2_O_2_] − 3.6912·[Polyphenols]^2^ − 6.465·[Polyphenols]·[Fe^3+^] + 1.0075·[Polyphenols]·[H_2_O_2_] − 3.3836·[Fe^3+^]^2^ − 0.94·[Fe^3+^]·[H_2_O_2_] − 6.9439·[H_2_O_2_]^2^

**Figure 9:**
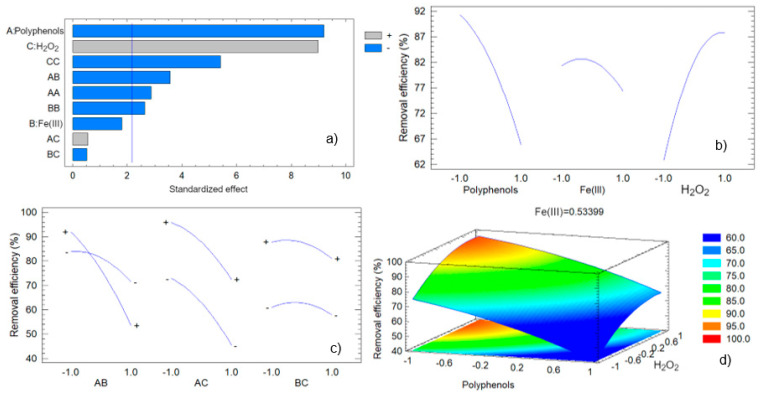
Graphical analysis of the experimental design corresponding to the removal of methylene blue: (**a**) Pareto factor plot; (**b**) main effects plot; (**c**) interactions plot; (**d**) response surface plot.

Figure 9 depicts the graphical analysis of the removal of methylene blue by the green-synthesized nZVI, which provides valuable insights into the effects of the operational variables and their interactions on the removal efficiency. The Pareto chart (Figure 9a) highlights the standardized effects of the factors and their interactions. The chart shows that hydrogen peroxide (C) has the most significant positive impact on the removal efficiency, whereas polyphenols (A) exert a significant negative effect. The interaction between polyphenols and iron (III) (AB) also appears prominently, indicating its substantial negative influence. These findings align with the estimated effects, confirming that hydrogen peroxide enhances removal efficiency, while polyphenols and their interaction with iron (III) reduce it. Interestingly, the quadratic effect of hydrogen peroxide leads to a negative influence on the removal efficiency. This latter is compatible with the fact that an excess of hydrogen peroxide leads to the generation of large amounts of hydroxyl radicals that may exert a self-scavenging effect, according to equation (6), as previously reported by our research group [64,65]:

(6) $$\cdot OH+H_{2}O_{2}\to HO_{2}\cdot +H_{2}O$$

The main effects plot (Figure 9b) illustrates the individual impact of each factor on the removal efficiency. It shows a clear negative trend for polyphenols (A), confirming that increasing the concentration of polyphenols decreases the removal efficiency, as indicated above. In contrast, hydrogen peroxide (C) displays a positive trend, where higher concentrations lead to improved removal efficiency. Iron (III) (B) has a minimal effect, as reflected in the nearly flat line.

The interaction plots (Figure 9c) provide a deeper understanding of the combined effects of the variables. The plot for the interaction between polyphenols and iron (III) (AB) reveals a significant negative interaction, suggesting that high levels of both these factors together reduce the removal efficiency more than when considered individually. The plots for the interactions between polyphenols and hydrogen peroxide (AC), and between iron (III) and hydrogen peroxide (BC), do not show strong interactions, indicating that their combined effects are less significant.

The response surface plot (Figure 9d), with Fe(III) (B) held constant at its optimal value, shows how the removal efficiency varies with polyphenols (A) and hydrogen peroxide (C). The plot reveals a saddle-like region indicating lower efficiency at higher polyphenol concentrations coupled with lower H_2_O_2_ concentration, reinforcing the negative impact of polyphenols and the positive effect of moderate concentration of hydrogen peroxide.

The plot of observed versus predicted values (Figure S4a, Supplementary Materials) demonstrates good agreement between the model’s predictions and the actual experimental data. The points closely align along the diagonal line, indicating that the model accurately predicts the removal efficiency. This high level of agreement validates the model’s reliability and robustness.

#### 3.3.2. Removal of Methyl Orange

For methyl orange, the statistical analysis revealed several notable differences compared to methylene blue. Polyphenols exhibited a negative impact on removal efficiency, with an effect estimate of −8.45454 (see Table S3, Supplementary Materials). Although less severe than the impact on methylene blue, this negative effect aligns with the tendency of organic compounds to inhibit nZVI catalytic activity by blocking active sites. Hydrogen peroxide showed a substantial positive effect (13.7804), consistent with its role in generating hydroxyl radicals crucial for dye degradation in Fenton-like processes. Interestingly, iron (III) had a positive effect (14.0834), enhancing removal efficiency, likely due to its role in catalyzing the Fenton-like reactions.

The ANOVA results (Table S4, Supplementary Materials) indicate that all the effects have *p*-values less than 0.05, confirming their statistical significance, which is worth noting. The standard error of the estimate is 1.2584, indicating a high level of model accuracy. The mean absolute error (MAE) is 0.7342, showing that the model’s predictions are, on average, very close to the actual values. The Durbin–Watson statistic is 1.9256 with a *p*-value of 0.1534, indicating no significant autocorrelation in the residuals and suggesting the model’s residuals are independent. These parameters collectively indicate a robust and reliable regression model for methyl orange removal, validated by a high R^2^ of 99.12% for the following equation:

Removal %(Methyl orange) = 85.4013 − 4.22727·[Polyphenols] + 7.0417·[Fe^3+^] + 6.89021·[H_2_O_2_] − 1.46081·[Polyphenols]^2^ − 2.22417·[Polyphenols]·[Fe^3+^] − 2.4233·[Polyphenols]·[H_2_O_2_] − 3.76669·[Fe^3+^]^2^ − 5.80297·[Fe^3+^]·[H_2_O_2_] − 4.21647·[H_2_O_2_]^2^

**Figure 10:**
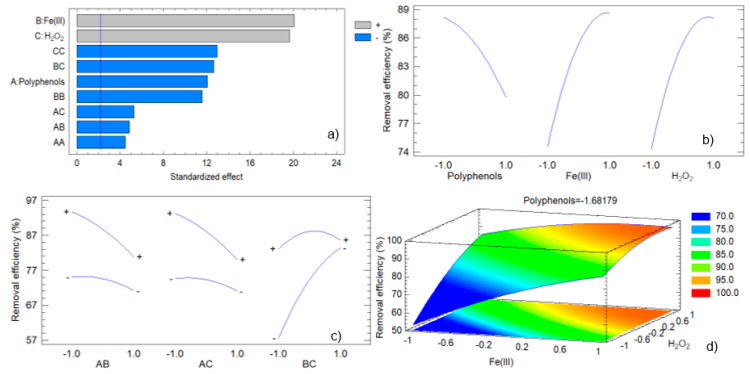
Graphical analysis of the experimental design corresponding to the removal of methyl orange: (**a**) Pareto factor plot; (**b**) Main effects plot; (**c**) Interactions plot; (**d**) Response surface plot.

Graphical analysis further supports these findings. The Pareto chart (Figure 10a) highlights the significant positive effects of hydrogen peroxide (C) and iron (III) (B), with polyphenols (A) having a less pronounced but still negative impact. Interaction effects, particularly between polyphenols and iron (III) (AB), are also significant, indicating complex dynamics that can affect efficiency.

The main effects plot (Figure 10b) shows that increasing polyphenol concentration decreases removal efficiency, while increasing hydrogen peroxide and iron (III) concentrations enhance it. Interaction plots (Figure 10c) reveal significant interactions between polyphenols and iron (III), suggesting that high concentrations of both reduce efficiency, potentially due to competitive adsorption or complex formation.

Response surface plots (Figure 10d) illustrate the optimal regions for high removal efficiency, emphasizing the critical balance between polyphenols and hydrogen peroxide concentrations. The observed versus predicted values plot (Figure S4b, Supplementary Materials) confirms the model’s accuracy, with points aligning closely along the diagonal line.

#### 3.3.3. Removal of Orange G

For orange G, the statistical analysis presents some differences from those observed for methylene blue and methyl orange. Polyphenols exhibited a minimal and statistically non-significant impact on the removal efficiency, with an effect estimate of −1.00515 (see Table S3, Supplementary Materials). This suggests that changes in polyphenol concentration do not significantly influence the efficiency of orange G removal. In contrast, hydrogen peroxide demonstrated a substantial positive effect (12.7022), indicating its crucial role in producing hydroxyl radicals necessary for effective dye degradation. Similarly, iron (III) showed a notable positive effect (11.1911), likely due to its role in catalyzing Fenton-like reactions.

The ANOVA results (Table S4, Supplementary Materials) confirmed the statistical significance of several effects, including the main effects of iron (III) and hydrogen peroxide, the quadratic term of the former, and the interaction between iron (III) and hydrogen peroxide. The model’s standard error of 2.7281 indicates good accuracy, while the mean absolute error (MAE) of 1.50523 suggests that the model’s predictions are generally close to the actual values. The Durbin–Watson statistic of 1.57596 with a *p*-value of 0.1627 indicates no significant autocorrelation in the residuals, affirming the model’s reliability. The high R^2^ value of 95.12% further supports the robustness of the regression model for orange G removal, so that the experimental results fit very well with the following equation:

Removal %(Orange G) = 91.055 − 0.502577·[Polyphenols] + 5.59557⋅[Fe^3+^] + 6.35108⋅[H_2_O_2_] + 1.42865⋅[Polyphenols]^2^ − 1.87654·[Polyphenols]·[Fe^3+^] − 1.07904⋅[Polyphenols]⋅[H_2_O_2_] −5.0003⋅[Fe^3+^]^2^ − 5.72841·[Fe^3+^]⋅[H_2_O_2_] − 3.3355⋅[H_2_O_2_]^2^

**Figure 11:**
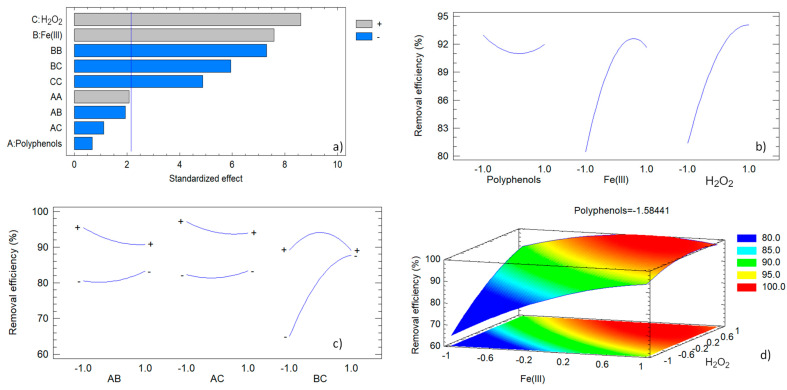
Graphical analysis of the experimental design corresponding to the removal of orange G: (**a**) Pareto factor plot; (**b**) main effects plot; (**c**) interactions plot; (**d**) response surface plot.

The graphical analysis corroborates these findings. The Pareto chart (Figure 11a) highlights the strong positive effects of hydrogen peroxide (C) and iron (III) (B), with polyphenols (A) showing a much smaller and non-significant impact. Notably, the interaction between iron (III) and hydrogen peroxide (BC) is significant, illustrating the complex interplay between these variables.

The main effects plot (Figure 11b) indicates that increasing the concentrations of iron (III) and hydrogen peroxide enhances removal efficiency, while polyphenols have a negligible impact. The interaction plots (Figure 11c) reveal significant synergy between iron (III) and hydrogen peroxide, indicating that their combined high concentrations lead to improved efficiency, possibly due to enhanced Fenton-like reactions.

Response surface plots (Figure 11d) illustrate optimal regions for high removal efficiency, emphasizing the importance of balancing iron (III) and hydrogen peroxide concentrations. The observed versus predicted values plot (Figure S4c, Supplementary Materials) demonstrates good agreement, confirming the model’s accuracy.

### 3.4. Optimization of the Dye Removal

A detailed analysis of the optimization process is vital for maximizing the efficiency of dye removal using the synthesized zero-valent iron nanoparticles (nZVI). In this section, the optimal conditions for dye removal are determined through a systematic approach, ensuring the highest possible degradation performance under varying operational parameters. The experimental design described in the previous subsection provides a robust statistical model, confirming the significant effects of polyphenols, Fe(III), and H_2_O_2_ on dye removal efficiency. The model predictions were in close agreement with experimental data, indicating the reliability of the optimized conditions. Next, to establish a reliable method for optimizing nZVI-based dye degradation processes, ensuring reproducibility and scalability, the optimization of the process was performed. Another important potentiality of experimental designs is their usefulness for determining the optimal values of operational variables to achieve a desired outcome (i.e., maximizing, minimizing, or hitting a target value of the response variable). In this study, our objective was to maximize the removal efficiencies of three dyes: methylene blue, methyl orange, and orange G. To achieve this, we set a target removal efficiency of 100% and identified the corresponding operational variable values.

Table 3 lists the coded and natural values of the operational variables required to achieve the total removal of each dye from water.

The data in Table 3 demonstrate that optimizing the three operational variables within the coded values range of −1 to 1 is feasible for achieving the desired removal efficiencies. This indicates the effectiveness of the experimental design approach. To validate the model’s predictions, we conducted three experiments under the conditions specified in Table 3. Theoretically, these conditions should result in complete dye removal.

The experimental results confirmed removal efficiencies of 100% for methylene blue and orange G, and 99.8% for methyl orange. These findings demonstrate that the model accurately predicts system behavior and that by precisely tuning the experimental conditions, complete removal of methylene blue and orange G, as well as nearly complete removal of methyl orange, can be achieved.

### 3.5. Some Insights into the Degradation Mechanism of Dyes

Analyzing the degradation mechanisms of dyes is crucial for understanding how the synthesized zero-valent iron nanoparticles (nZVI) interact with and break down these pollutants. In this section, some insights into the chemical pathways and processes involved in the degradation of different dyes are provided, shedding light on the roles of adsorption, radical generation, and other relevant factors. The degradation of dyes in aqueous systems using zero-valent iron nanoparticles (nZVI) synthesized from waste tea presents a promising approach for environmental remediation. However, determining the precise degradation pathways in such systems is extremely challenging due to their inherent complexity. In addition to the dye molecules themselves, the system contains a wide variety of organic compounds, particularly the diverse polyphenols present in the tea waste extract used during the synthesis of nZVI. These polyphenols, while playing a crucial role in both the reduction of iron salts and the stabilization of nanoparticles, introduce a multitude of reactive species and intermediates during the degradation process. The interactions between these organic molecules and the reactive species generated by nZVI and hydrogen peroxide create a highly complex network of reactions, making it difficult to pinpoint exact degradation pathways.

The complexity of the system makes it extremely difficult, if not nearly impossible, to follow the degradation pathway through the conventional use of HPLC-MS techniques. Furthermore, radical-mediated reactions, such as those driven by hydroxyl radicals, tend to be very fast. This rapid reaction rate complicates the quenching of the reaction at preset time intervals, making it challenging to capture and analyze intermediate products accurately.

The degradation of dyes by nZVI involves a complex interplay of chemical reactions, where nZVI, polyphenols from the tea extract, and hydrogen peroxide (H₂O₂) each play critical roles.

Firstly, the nZVI reported in this work exhibits a large surface area, which makes it an effective adsorbent for dye molecules. The adsorption of dyes onto the surface of nZVI can occur as the sole removal pathway or serve as a convenient preliminary step that concentrates the dye molecules at the surface, making them more susceptible to subsequent degradation by hydroxyl radicals. The large surface area of nZVI allows for a high adsorption capacity, enabling it to capture a significant amount of dye from the aqueous solution. This adsorption process not only directly reduces the dye concentration in water but also facilitates more efficient degradation by bringing the dye molecules into close proximity with the reactive sites on the nZVI surface. Desorption experiments revealed that for methylene blue, adsorption is the predominant removal mechanism, as the dye was re-released into the solution. Conversely, for orange G and methyl orange, the lack of desorption indicates that these dyes are primarily degraded rather than merely adsorbed, as discussed below.

For azo dyes like orange G and methyl orange, the hydroxyl radicals primarily target the azo bond (–N=N–), leading to its cleavage. The cleavage of this bond results in the formation of aromatic amines, which are typically more susceptible to further oxidative degradation. The key reaction is:

(7) $$\cdot OH+R-N=N-R^{\prime }\to R-NH_{2}+R^{\prime }-NH_{2}$$

The resulting aromatic amines can then undergo further degradation to smaller, less toxic compounds, ultimately leading to the mineralization of the dye.

The degradation of methylene blue (MB) involves a series of oxidation reactions initiated by hydroxyl radicals. The hydroxyl radicals attack the sulfur and nitrogen atoms within the MB molecule, leading to the breakdown of the chromophore structure. The degradation of MB can follow several pathways, including (i) demethylation, consisting of the removal of methyl groups attached to the nitrogen atoms, leading to the formation of intermediate products like azure A, azure B, and azure C; and (ii) cleavage of the central phenothiazine ring structure, that can be opened by hydroxyl radicals, leading to the formation of smaller aromatic compounds.

The intermediate compounds resulting from these processes can be further oxidized to simpler, less colored, and less toxic organic molecules, eventually resulting in complete mineralization to CO₂, H₂O, and inorganic ions.

Polyphenols extracted from waste tea serve a dual role in the synthesis and stabilization of nZVI, as well as in the dye degradation process. During synthesis, polyphenols act as reducing agents, converting Fe(III) ions to Fe(0), thus facilitating the formation of nZVI:

(8) $${Fe}^{3+}+Polyphenol\to {Fe}^{0}+Oxidizedpolyphenol$$

The antioxidant properties of polyphenols also help stabilize nZVI by preventing excessive oxidation and aggregation. 

During the dye degradation process, polyphenols can influence the redox cycling between Fe(II) and Fe(III), potentially enhancing the generation of hydroxyl radicals:

(9) $${Fe}^{3+}+Polyphenol\to {Fe}^{2+}+Oxidizedpolyphenol$$

This interaction supports the sustained production of reactive species necessary for effective dye degradation.

Finally, hydrogen peroxide is crucial in the Fenton and Fenton-like reaction catalyzed by nZVI. When H₂O₂ is introduced into the system, it reacts with Fe(II) to produce hydroxyl radicals:

(10) $${Fe}^{2+}+H_{2}O_{2}\to {Fe}^{3+}+\cdot OH+OH^{-}$$

The hydroxyl radicals generated are highly reactive and capable of breaking down complex dye molecules into smaller, less harmful compounds:

(11) OH+Dye→Degradedproducts

The iron oxide or oxohydroxide layer formed on the nZVI surface due to partial oxidation can also contribute to the catalytic activity, as it may participate in the decomposition of H₂O₂, further generating reactive species:

(12) $$2FeOOH+H_{2}O_{2}\to {Fe}_{2}O_{3}+\cdot OH+OH^{-}+H_{2}O$$

According to all of these, the combined action of nZVI, polyphenols, and H₂O₂ results in a synergistic effect that enhances dye degradation. Polyphenols not only stabilize nZVI but also promote the catalytic generation of reactive species, while H₂O₂ ensures a continuous supply of hydroxyl radicals. Together, these components create a highly efficient system for breaking down stubborn dye pollutants in water.

## 4. Conclusions

In this study, polyphenols extracted from tea leaf waste have been used for the green synthesis of zero-valent iron nanoparticles (nZVI), demonstrating a sustainable approach to environmental remediation. The tea waste extract, rich in antioxidant polyphenols such as catechin, gallocatechin, epicatechin, and epigallocatechin-3,5-digallate, was effectively utilized to reduce iron salts, forming nZVI.

Comprehensive characterization of the tea waste extract and the synthesized nZVI affirmed their suitability for practical applications. The HPLC and Folin–Ciocalteu assay confirmed the high polyphenol content, which played a pivotal role as reducing agents in the nZVI synthesis. The nZVI displayed favorable physiochemical properties, including a high specific surface area and significant mesoporosity, as revealed by gas adsorption of N2 at 77 K. SEM and TEM images showed a rough, aggregated morphology with particle sizes below 50 nm. FT-IR spectroscopy identified the presence of functional groups from both polyphenols and iron oxides, while XRD confirmed the formation of zero-valent iron and iron oxides. Thermal analyses, including TG and TPR, demonstrated the stability and reduction behavior of the nZVI, supporting a core-shell structure with a Fe(0) core surrounded by iron oxides.

The in situ generated nZVI, when combined with hydrogen peroxide, facilitated the efficient removal of dyes (methylene blue, methyl orange, and orange G) via a hetero-catalytic Fenton process. The experimental design and response surface methodology optimized the operational variables, achieving removal efficiencies of over 99.5% for all tested dyes, with complete removal for methylene blue and orange G, and 99.8% for methyl orange. These results validate the model’s predictive accuracy and the effectiveness of the green-synthesized nZVI.

This research underscores the dual benefits of utilizing antioxidant-rich food by-products for environmental applications. The polyphenols from tea waste provide a sustainable source of reducing agents for nZVI synthesis, so this approach not only contributes to waste valorization but also may offer a cost-effective and eco-friendly solution for addressing environmental pollution. Nonetheless, one of the key issues in waste valorization is the logistics and expenses associated with the collection and transportation of the waste material. Hence, while the method holds promise for its environmental benefits, addressing the economic feasibility on a larger scale is crucial. Future research and pilot studies focusing on the economic analysis and optimization of waste collection and processing will be essential to validate the cost-effectiveness of this approach.

Also, while this study demonstrates the effective removal of dyes from wastewater using nZVI, the potential release and environmental impact of these nanoparticles must be carefully considered. In future studies, the fate and toxicity of nZVI and its byproducts in the treated water should be thoroughly investigated. This will include detailed assessments of the persistence, bioaccumulation, and potential harmful effects on aquatic organisms and ecosystems. Additionally, strategies for the safe recovery and reuse of nZVI will be explored to minimize any adverse environmental impact.

In summary, it can be concluded that the use of food by-products such as tea waste for the synthesis of functional materials like nZVI presents a promising strategy for sustainable environmental technologies, although its cost-effectiveness and potential toxic effects should be investigated in the future.

## Figures and Tables

**Table 1:** Experimental matrix and removal efficiencies for the three target pollutants.

Experiment	Coded Values			Removal Efficiency (%)		
	Polyphenols	Fe(III)	H_2_O_2_	Methylene Blue	Methyl Orange	Orange G
1	−1	−1	1	91.76	86.68	93.72
2	0	0	0	82.12	85.67	90.72
3	−1.68179	0	0	94.4	87.45	93.57
4	0	0	0	82.54	86.89	90.96
5	0	0	0	83.96	84.96	91.52
6	0	0	0	83.22	83.63	90.96
7	−1	1	1	92.03	93.47	92.22
8	0	0	0	83.75	86.83	91.60
9	0	1.68179	0	66.04	85.72	88.90
10	1.68179	0	0	41.32	73.47	93.62
11	0	−1.68179	0	71.42	62.15	61.92
12	0	0	0	79.74	83.82	90.04
13	1	1	1	64.46	75.80	86.13
14	0	0	0	78.69	83.94	92.62
15	1	−1	−1	58.77	57.28	72.65
16	1	−1	1	81.01	77.54	92.02
17	0	0	0	81.8	86.45	90.51
18	1	1	−1	36.94	78.39	86.56
19	−1	−1	−1	64.51	56.36	66.92
20	0	0	0	85.01	86.70	91.08
21	0	0	1.68179	82.01	84.37	92.08
22	−1	1	−1	77.58	86.73	91.45
23	0	0	−1.68179	35.31	60.96	68.16

**Table 2:** Semi-quantitative analysis of the crystalline phases of the nZVI sample.

Compound	Formula	Percent	Crystal System	Space Group	a	B	C	α	β	γ
Goethite	FeOOH	31.41	Tetragonal	I4/m	10.48	10.48	3.02	90	90	90
α-Iron	Fe	44.73	Cubic	Im-3m	2.87	2.87	2.87	90	90	90
Hematite	Fe_2_O_3_	23.86	Tetragonal	P4_3_2_1_2	8.34	8.34	25.02	90	90	90

**Table 3:** Coded and natural values of operational variables for maximizing dye removal efficiencies, and experimental confirmation of the maxima.

Variable	Methylene Blue		Methyl Orange		Orange G	
	Coded	Natural	Coded	Natural	Coded	Natural
[Polyphenols] (mg·L^−1^)	−0.62116	175	−0.68179	165	−0.58441	181
[Fe^3+^] (M)	0.53399	2.64 × 10^−2^	0.914226	3.09 × 10^−2^	0.731064	2.87 × 10^−2^
[H_2_O_2_] (M)	0.93049	1.55 × 10^−2^	0.671226	1.40 × 10^−2^	0.795279	1.47 × 10^−2^
**Removal Efficiency under Optimal Conditions**						
	**Methylene blue**		**Methyl orange**		**Orange G**	
	100%		98%		100%	

## Data Availability

The raw data supporting the conclusions of this article will be made available by the authors on request.

## Supplementary Materials

The following supporting information can be downloaded at: https://www.mdpi.com/article/10.3390/antiox13091059/s1, Table S1. Determination of the standard line of gallic acid; Table S2. Coded and real values of the operational variables and experimental design matrix; Table S3. Estimated effects for the removal of the three dyes; Table S4. Results of the analysis of variance (ANOVA); Figure S1. Standard line for the quantification of gallic acid in solution; Figure S2. Experimental setup for the synthesis of nZVI. Figure S3. (a,b) SEM images of nZVI samples prepared using NaBH_4_ as the reducing agent. (c,d) SEM images of nZVI samples prepared using tea waste extract as the reducing agent. Figure S4. Observed versus predicted values plots: Methylene blue (a); Methyl orange (b); orange G (c).
