# Supplementary material for: Valorizing Tea Waste: Green Synthesis of Iron Nanoparticles for Efficient Dye Removal from Water
Source: Antioxidants (Basel). 2024 Aug 30;13(9):1059. doi: 10.3390/antiox13091059 (PMC11429485; doi:10.3390/antiox13091059)
Supplement: Supplementary file 1 [file antioxidants-13-01059-s001.zip › antioxidants-3141374-supplementary.pdf]

Valorizing Tea Waste: Green Synthesis of Iron Nanoparticles for Efficient Dye Removal from Water

Cristina Rodríguez-Rasero, María F. Alexandre-Franco, Carmen Fernández-González, Vicente Montes-Jiménez and Eduardo M. Cuerda-Correa*

Departamento de Química Orgánica e Inorgánica, Facultad de Ciencias, Universidad de Extremadura, Avenida de Elvas s/n, 06006-Badajoz, España

SUPPLEMENTARY MATERIAL

Table S1. Determination of the standard line of gallic acid

Concentration (mg L^{-1})	Concentration ($\cdot 10^5$M)	Absorbance
4	0.884	0.18
6	1.33	0.273
8	1.77	0.363
10	2.21	0.452
12	2.65	0.543
14	3.09	0.635
16	3.54	0.722
18	3.98	0.813
20	4.42	0.902

Table S2. Coded and real values of the operational variables and experimental design matrix.

Variable	Coded value				
	**-1.68179**	**-1**	**0**	**1**	**1.68179**
**Polyphenols ($mg \cdot L^{-1}$)**	0	113	278	443	556
**Fe (III) (M)**	0	$8.1 \cdot 10^{-3}$	$2.0 \cdot 10^{-2}$	$3.2 \cdot 10^{-2}$	$4.0 \cdot 10^{-2}$
**H_2O_2 (M)**	0	$4.1 \cdot 10^{-3}$	$1.0 \cdot 10^{-2}$	$1.6 \cdot 10^{-2}$	$2.0 \cdot 10^{-2}$

Table S3. Estimated effects for the removal of the three dyes.

Effect	Methylene blue		Methyl orange		Orange G	
	**Estimated**	**V.I.F.**	**Estimated**	**V.I.F.**	**Estimated**	**V.I.F.**
A:Polyphenols	-25.4773	1.0	-8.45454	1.0	-1.00515	1.0
B:Fe(III)	-4.99208	1.0	14.0834	1.0	11.1911	1.0
C:H_2O_2	24.8959	1.0	13.7804	1.0	12.7022	1.0
AA	-7.3824	1.00009	-2.92162	1.00009	2.8573	1.00009
AB	-12.93	1.0	-4.44835	1.0	-3.75308	1.0
AC	2.015	1.0	-4.8466	1.0	-2.15808	1.0
BB	-6.76721	1.00009	-7.53338	1.00009	-10.0006	1.00009
BC	-1.88	1.0	-11.606	1.0	-11.4568	1.0
CC	-13.8878	1.00009	-8.43293	1.00009	-6.67099	1.00009

Note: The variance inflation factor (V.I.F.) in a factorial, central, composite, orthogonal and rotatable design indicates the degree of multicollinearity among the predictor variables, with higher values suggesting greater correlation and potential issues with the reliability of the regression coefficients. For a perfectly orthogonal design, all factors would equal 1. Factors of 10 or more are typically interpreted as indicative of serious collinearity among the effects.

Table S4. Results of the analysis of variance (ANOVA)

Factor	Methylene blue	Methyl orange	Orange G
	p-value	p-value	p-value
A:Polyphenols	**0.0000**	**0.0000**	0.5079
B:Fe(III)	0.0948	**0.0000**	**0.0000**
C:H_2O_2	**0.0000**	**0.0000**	**0.0000**
AA	**0.0130**	**0.0006**	0.0571
AB	**0.0034**	**0.0003**	0.0737
AC	0.5872	**0.0001**	0.2835
BB	**0.0206**	**0.0000**	**0.0000**
BC	0.6122	**0.0000**	**0.0000**
CC	**0.0001**	**0.0000**	0.5079
	**R^2** = 94.56 % ; **Standard error** = 5.12; **Mean Absolute Error** = 2.97; **Durbin-Watson** = 1.88 (P=0.4216)	**R^2** = 99.12 % ; **Standard error** = 1.30; **Mean Absolute Error** = 0.88; **Durbin-Watson** = 2.24 (P=0.7685)	**R^2** = 95.12 % ; **Standard error** = 2.73; **Mean Absolute Error** = 1.51; **Durbin-Watson** = 1.58 (P=0.1627)

Note: Factors exerting a statistically significant influence on the dye removal efficiency are shown in bold.

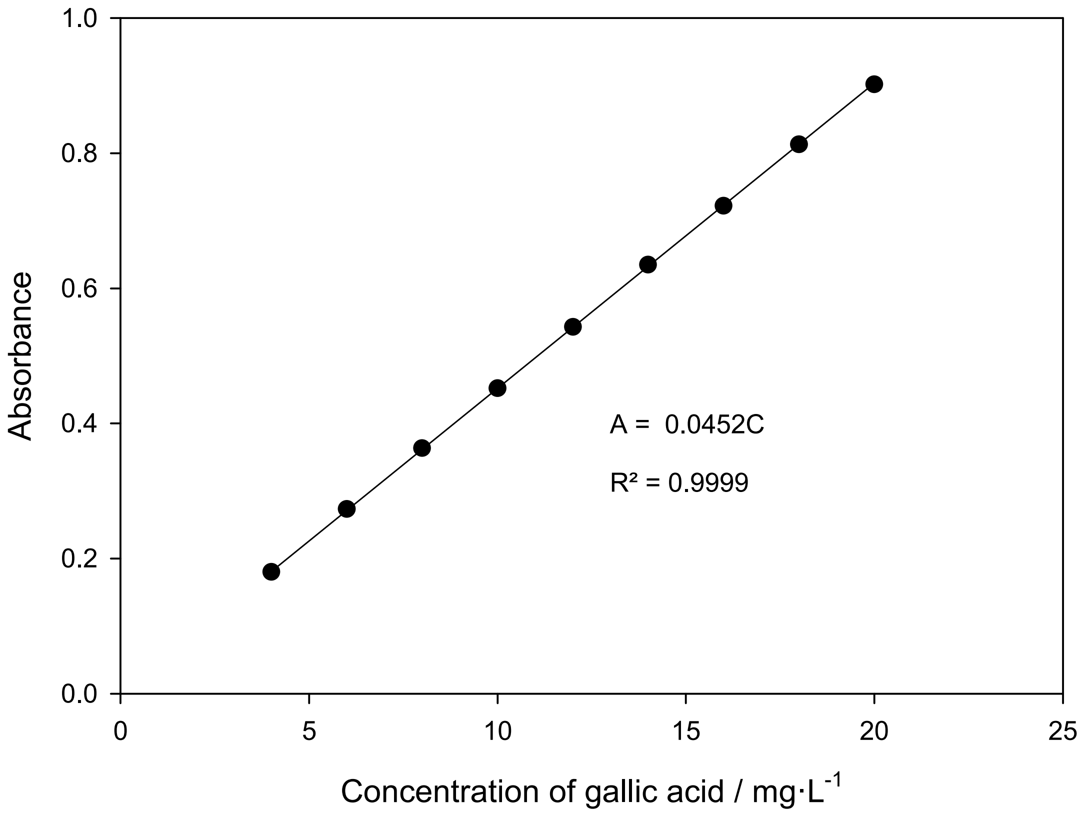

Figure S1. Standard line for the quantification of gallic acid in solution.

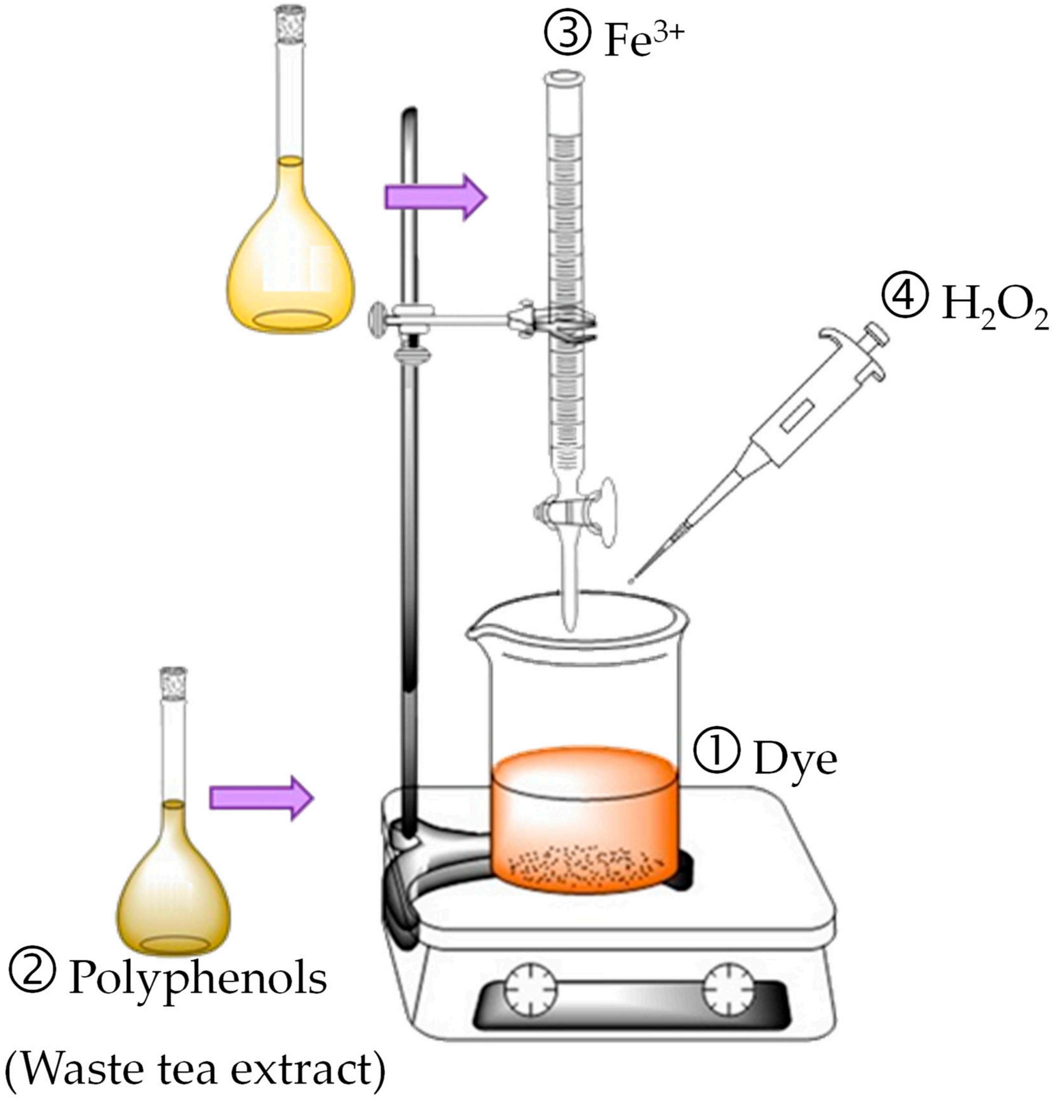

Figure S2. Experimental setup for the synthesis of nZVI.

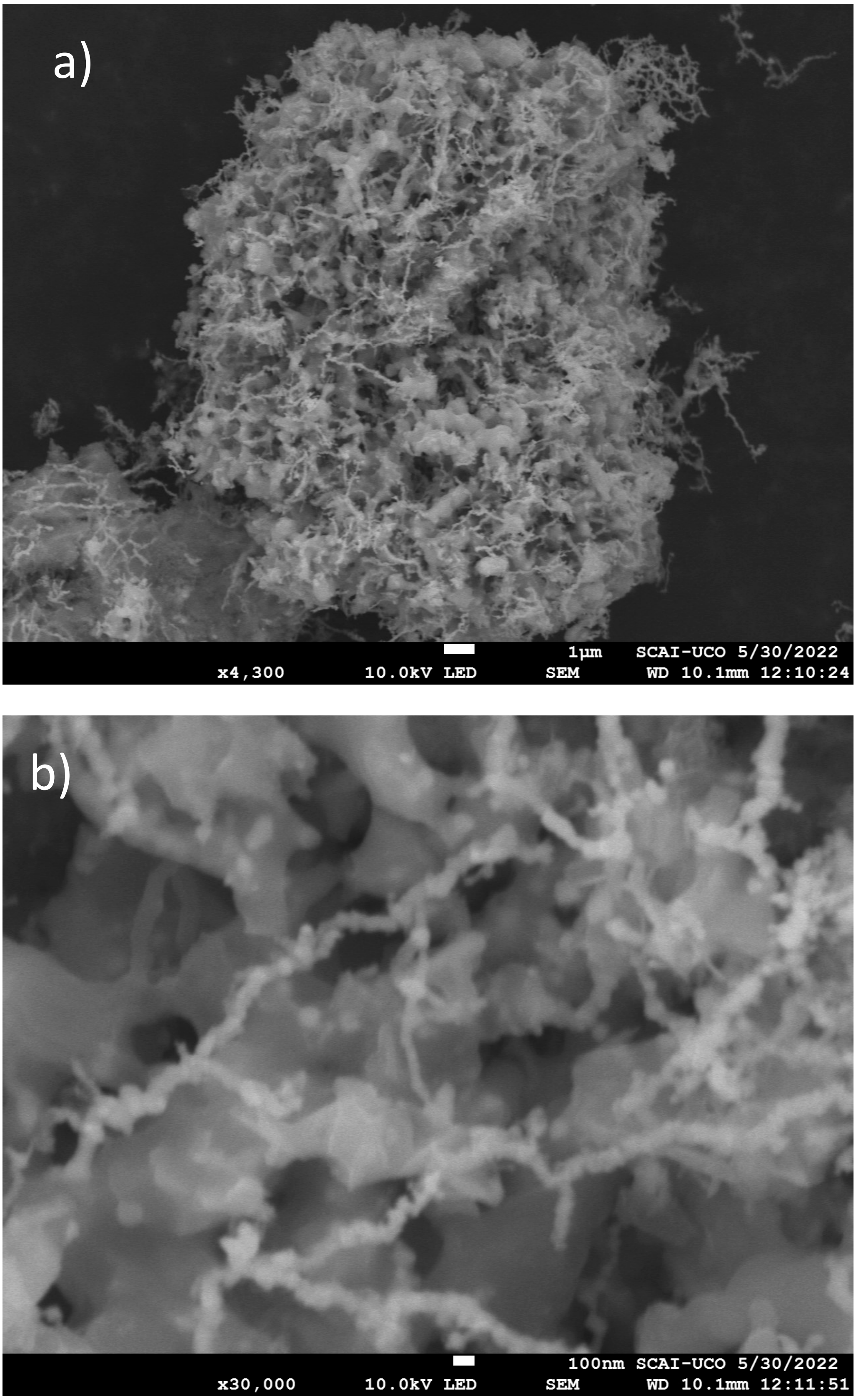

Figure S3. (a,b) SEM images of nZVI samples prepared using $NaBH_4$ as the reducing agent.

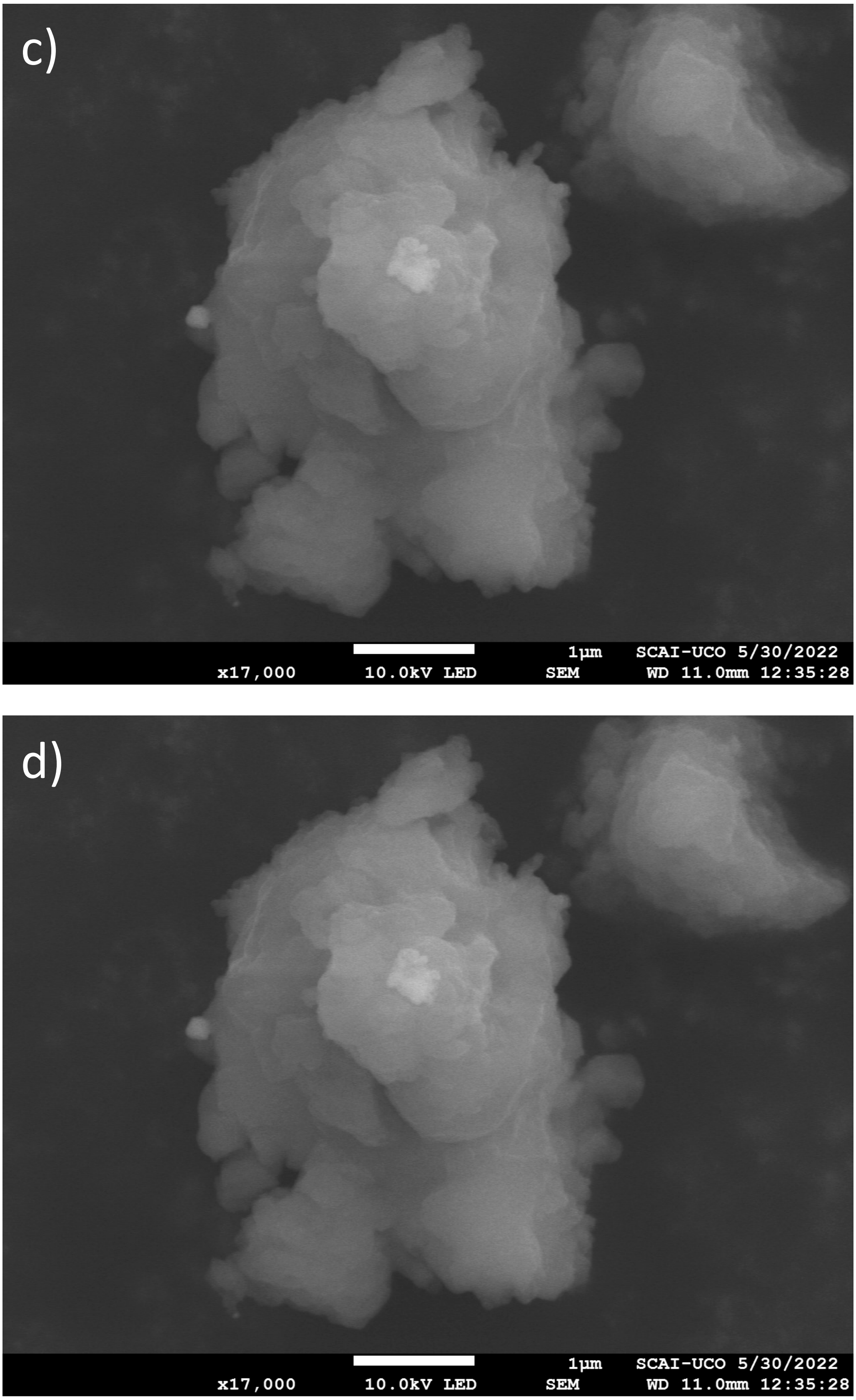

Figure S3. (c,d) SEM images of nZVI samples prepared using tea waste extract as the reducing agent.

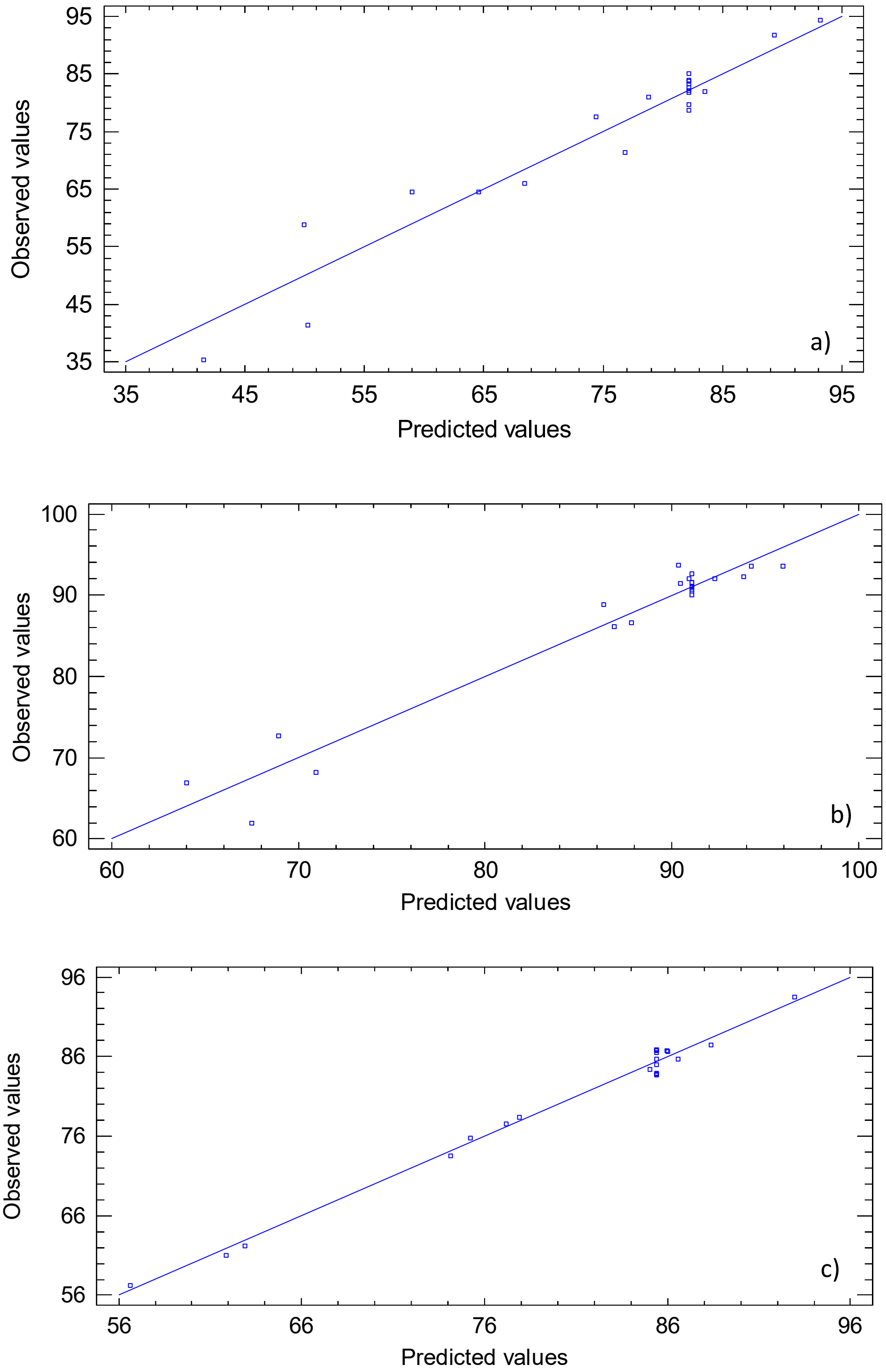

Figure S4. Observed versus predicted values plots: Methylene blue (a); Methyl orange (b); Orange G (c).
